# With Appreciation

**DOI:** 10.1002/jcsm.70169

**Published:** 2025-12-19

We thank the following individuals who served as manuscript reviewers for The Journal of Cachexia, Sarcopenia and Muscle (JCSM) in 2025.

We highly appreciate their efforts.

Fair, conscientious and timely peer reviews contribute to the success of JCSM.

The Editors.

**7 and more reviews**

Hazim Alhiti

Djandan Tadum Arthur Vithran

Amy Attaway

Jing‐Fu Bao

Vickie Baracos

Patricia Batista

Richard Bohannon

Starling Emerald Bright David

Toby Chambers

An‐Chih Chang

Liang‐Kung Chen

Xian Wu Cheng

Paola Costelli

Yaoshan Dun

Jens Fielitz

Wie Gao

Mikel García Aguirre

Denis Guttridge

Yoshitaka Hashimoto

Satoshi Ikeda

Masaaki Konishi

Gopal Nambi

Masatsugu Okamura

Luciana Pereira

Xinran Qi

Leonardo Roever

Megan Rosa‐Caldwell

Ishan Roy

Kunihiro Sakuma

Gaetano Santulli

Takayoshi Sasako

Hiroshi Shamoto

Tingting Yang

Hang Yin

Yoshihiro Yoshimura

Silke Zimmermann

**5–6 reviews**

Takashi Abe

Sue Acreman

Volker Adams

Belgin Akan

Matthew Alexander

Alejandro Álvarez Bustos

Hamzah Amin

Jann Arends

Christopher Axelrod

Mara Barone

Catherine Bellissimo

Emanuele Berardi

Matthew Bubak

Pierre Carlier

Tai‐Heng Chen

Meritxell Espino Guarch

Jinghua Gu

Joo Young Huh

Dandan Jia

Andy Khamoui

Il‐Young Kim

Frederick Koh

Ashok Kumar

Saurabh Mishra

Masaki Mogi

Ryan Montalvo

Christopher Nelke

Erik Niklasson

Ichizo Nishino

Jesus Perez‐Camiña

Terence Ryan

Akihiro Sakuyama

Ryosuke Sato

Ming Yang

Mee‐Sup Yoon

Rachel Zeng

**3–4 reviews**

Luis M Alegre

Anwar Ali

Stephen Alway

Ran An

Elia Angelino

Óscar Arellano‐Pérez

Paige Arneson‐Wissink

Martin Bahls

Felix Barajas

Joshua Barzilay

Yuri Battaglia

Philipp Baumert

Zyta Beata

Pascal Bernatchez

Bijoya Bhattacharjee

Jan Bilski

Jose‐Ramon Blanco

Philip Bonomi

Marina Bouche

Mia Brath

Jeffrey Brault

Danna Breen

Jacob Brown

Guoqi Cai

Donnie Cameron

Daniel Cannon

Luka Cavka

Yueqi Chen

Liangkai Chen

Joe Chiles

Gabriela Cipolli

Aleksandar Cirovic

Andrew Coats

Rachel Cooper

Flavio Da Silva

Lunzhi Dai

Srinivasan Dasarathy

Torsten Diekhoff

Shengguang Ding

Wolfram Doehner

Hervé Dubouchaud

Bradley Elliott

Mert Eşme

Rafaela Espírito Santo

Karyn Esser

William Evans

Jens Fielitz

Guilherme Fonseca

Jordan Fuqua

Lin Gao

Shuguang Gao

Tania Garfias‐Veitl

Eduardo Godoy

Jie Guo

Jiong Jiong Guo

Tewfik Hamidi

Guillaume Henin

Zachary Hettinger

Zhaoyong Hu

Shih Tsung Huang

Jung Keun Hyun

Tatsuro Inoue

Wei Ji

Honglei Ji

Hua Jiang

Jong‐Sun Kang

Stephan Kersting

Nam Hoon Kim

Chris Kim

Xingxing Kong

Leonidas Koniaris

Maciej Lalowski

Alberto Lana

Wei‐Ju Lee

Christoph Lepper

Paul Lewandowski

Yi‐Ping Li

Lei Li

Jin Li

Guoping Li

Teerin Liewluck

Fangchao Liu

Xinran Ma

Marwan Mannaa

Hamish McAuley

Clare McKeaveney

Paulo Mesquita

Pablo Molina

Xin Ni

Stephan Oehme

Takuro Okamura

Cameron Oswalt

Michael Paris

Joon Y Park

Traci Parry

Stuart Phillips

Carla Prado

Feodor Price

S Russ Price

Konstantinos Prokopidis

Shauna Rakshe

Andrea David Re Cecconi

Heitor Ribeiro

Vanina Romanello

Joseph Rupert

Irena Sarc

Fabio Sarto

Dalton Schiessel

Paula Schmidt Azevedo Gaiolla

Martina Schweiger

Mitsuhiro Shimura

Vanessa Stadlbauer

Barbara Strasser

Kristy Swiderski

Erin Talbert

Malte Tiburcy

Andrea Ticinesi

Peter Tickle

Michael Toth

Alessio Turco

Matthias Türk

Flavio Vieira

Kenneth Weber

Simon Wing

Jean Woo

Wouter Worp

Tong Wu

Lingyan Xu

Koichi Yamamoto

Tingting Yang

Kang Yu

Qingchun Zeng

Hongmei zhang

QuanZhang

